# Supplementary material for: Prenatal influenza vaccination and allergic and autoimmune diseases in childhood: A longitudinal, population-based linked cohort study
Source: PLoS Med. 2022 Apr 5;19(4):e1003963. doi: 10.1371/journal.pmed.1003963 (PMC9017895; doi:10.1371/journal.pmed.1003963)
Supplement: S1 Table — ICD-10-AM, International Statistical Classification of Diseases and Related Health Problems, Tenth Revision, Australian Modification. (DOCX) [file pmed.1003963.s002.docx]

**S1 Table. ICD-10-AM diagnosis codes used to identify allergic or autoimmune diseases, and all-cause injuries, and frequency of outcomes by data source.**

| **Outcome** | | | | **ICD-10-AM or Symptom Code** | **Both data sources^a,b^**  **(n)** | **Hospital inpatient admission (HMDC)^a^**  **(n)** | **Emergency department episode (EDDC)^b^**  **(n)** |
| --- | --- | --- | --- | --- | --- | --- | --- |
| Allergic or autoimmune diseases | | | | Composite of all codes listed below: | 8,568 | 3,819 | 6,074 |
|  | Allergic diseases | | |  | 8,416 | 3,674 | 6,017 |
|  |  | Allergic rhinitis | | J30, J45.0 | 148 | 35 | 114 |
|  |  | Asthma and wheezing | | J45, J46, SQA00*, SQAA0*, R06.2, CH000* | 3,757 | 3,078 | 1,519 |
|  |  |  | Asthma | J45, J46, SQA00*, SQAA0* | 1,556 | 637 | 1,276 |
|  |  |  | Wheezing | R06.2, CH000* | 2,906 | 2,732 | 290 |
|  |  | Hypersensitivity pneumonitis due to organic dust | | J67 | 0 | 0 | 0 |
|  |  | Allergic gastroenteritis | | K52.2 | 82 | 82 | 0 |
|  |  | Atopic dermatitis | | L20 | 270 | 51 | 224 |
|  |  | Allergic dermatitis | | L23 | 713 | 19 | 694 |
|  |  | Urticaria | | L50 | 2,994 | 229 | 2,848 |
|  |  | Anaphylaxis | | T78.0, T78.2, T78.4, T80.5, T88.1, T88.6, T88.7 | 1,157 | 294 | 1,041 |
|  |  | Angioneurotic oedema | | T78.3 | 121 | 21 | 105 |
|  | Autoimmune diseases | | |  | 174 | 160 | 61 |
|  |  | Idiopathic thrombocytopenic purpura | | D69.3 | 33 | ** | <5 |
|  |  | Diabetes mellitus | | E10-14 | 54 | 48 | 51 |
|  |  | Addison’s disease | | E27.1 | <5 | <5 | 0 |
|  |  | Multiple sclerosis | | G35 | 0 | 0 | 0 |
|  |  | Vasculitis | | I77.6, L95, M30.1-M30.2, M31.1-M31.2,  M31.4-M31.9 | 9 | ** | <5 |
|  |  | Ulcerative colitis and Crohn's disease | | K50-K51 | 7 | ** | <5 |
|  |  | Inflammatory liver diseases | | K75 | 9 | 5 | 5 |
|  |  | Coeliac disease | | K90.0 | 40 | 40 | 0 |
|  |  | Reactive arthropathy | | M02.9 | 0 | 0 | 0 |
|  |  | Juvenile arthritis | | M08 | 21 | 21 | 0 |
|  |  | Dermatopolymyositis | | M33 | 0 | 0 | 0 |
|  |  | Sjögren’s disease | | M35.0 | 0 | 0 | 0 |
|  |  | Lupus | | L93, M32 | 0 | 0 | 0 |
|  |  | Localized connective tissue disorders | | L94 | 0 | 0 | 0 |
| Negative control condition: all-cause injury admission | | | | Composite of all codes listed below: | 21,730 | 3,528 | 20,898 |
|  | Head | | | S01-S09 | 14,466 | 2,347 | 13,786 |
|  | Neck | | | S11-S19 | 200 | 30 | 179 |
|  | Thorax | | | S21-S29 | 106 | 53 | 64 |
|  | Abdomen, lower back, lumbar spine and pelvis | | | S31-S39 | 365 | 63 | 315 |
|  | Shoulder and upper arm | | | S41-S49 | 1,005 | 172 | 932 |
|  | Elbow and forearm | | | S51-S59 | 3,481 | 189 | 3,407 |
|  | Wrist and hand | | | S61-S69 | 1,868 | 473 | 1,759 |
|  | Hip and thigh | | | S71-S79 | 254 | 122 | 240 |
|  | Knee and lower leg | | | S81-S89 | 1,120 | 99 | 1,077 |
|  | Ankle and foot | | | S91-S99 | 1,541 | 154 | 1,478 |
|  | Multiple site | | | ABD* | 0 | - | 0 |
|  | Chest | | | ABE* | 0 | - | 0 |
|  | Head | | | ABF* | 0 | - | 0 |
|  | Limb | | | ABG* | 0 | - | 0 |
|  | Burn | | | ABH* | 0 | - | 0 |
|  | Laceration | | | ABI* | 0 | - | 0 |
|  | Facial | | | ABJ* | 0 | - | 0 |
|  | Eye | | | ABK* | 0 | - | 0 |
|  | Bite | | | ABL* | 0 | - | 0 |
|  | Sexual | | | ABM* | 0 | - | 0 |
|  | Insect bite | | | ABN* | 0 | - | 0 |
|  | Nil | | | ABO* | 0 | - | 0 |
| Abbreviations: EDDC, Emergency Department Data Collection; HMDC, Hospital Morbidity Data Collection; ICD-10-AM, International Statistical Classification of Diseases and Related Health Problems, Tenth Revision, Australian Modification.  ^a^ Principal and all additional diagnoses fields were used to classify outcomes.  ^b^ Principal diagnosis fields, and symptom fields were used to classify outcomes.  * Symptom code used if principal diagnosis field is missing (EDDC only).  ** In accordance with privacy and confidentiality guidelines by the WA Data Linkage Branch, secondary suppression was used to prevent suppressed cells (<5) from being recalculated through subtraction. | | | | | | | |
