# Supplementary material for: Prenatal influenza vaccination and allergic and autoimmune diseases in childhood: A longitudinal, population-based linked cohort study
Source: PLoS Med. 2022 Apr 5;19(4):e1003963. doi: 10.1371/journal.pmed.1003963 (PMC9017895; doi:10.1371/journal.pmed.1003963)
Supplement: S2 Table — (DOCX) [file pmed.1003963.s003.docx]

**S2 Table. Risk of allergic or autoimmune diseases associated with prenatal exposure to seasonal inactivated influenza vaccine among children <5 years of age who were born at term (>37 weeks gestational age), by trimester of prenatal vaccination.**

|  | | **Unexposed to seasonal influenza vaccine during pregnancy**  **(N = 102,974)** | **Exposed to seasonal influenza vaccine during pregnancy**  **(N = 13,509)** | **Trimester of vaccine exposure** | | |
| --- | --- | --- | --- | --- | --- | --- |
|  |  |  |  | **First trimester**  **(N = 2,600)** | **Second trimester**  **(N = 5,117)** | **Third trimester**  **(N = 5,792)** |
| *Allergic or autoimmune disease* | | | | | | |
|  | Cases, n (%) | 6,955 (6.8) | 841 (6.2) | 155 (6.0) | 361 (7.1) | 325 (5.6) |
|  | Unweighted HR (95% CI) | 1 [Reference] | 1.05 (0.97 to 1.12) | 0.99 (0.85 to 1.16) | 1.12 (1.00 to 1.24) | 1.00 (0.90 to 1.12) |
|  | Weighted aHR (95% CI)^a^ | 1 [Reference] | 1.03 (0.95 to 1.11) | 0.96 (0.81 to 1.14) | 1.10 (0.98 to 1.23) | 0.99 (0.88 to 1.12) |
| *Allergic disease* | | | | | | |
|  | Cases, n (%) | 6,835 (6.6) | 829 (6.1) | 153 (5.9) | 357 (7.0) | 319 (5.5) |
|  | Unweighted HR (95% CI) | 1 [Reference] | 1.05 (0.97 to 1.13) | 1.00 (0.85 to 1.17) | **1.12 (1.01 to 1.25)** | 1.00 (0.89 to 1.12) |
|  | Weighted aHR (95% CI)^a^ | 1 [Reference] | 1.03 (0.95 to 1.11) | 0.97 (0.82 to 1.15) | 1.10 (0.99 to 1.24) | 0.99 (0.87 to 1.11) |
| *Asthma diagnosis or wheezing* | | | | | | |
|  | Cases, n (%) | 2,977 (2.9) | 338 (2.5) | 60 (2.3) | 145 (2.8) | 133 (2.3) |
|  | Unweighted HR (95% CI) | 1 [Reference] | 1.02 (0.91 to 1.14) | 0.92 (0.72 to 1.17) | 1.07 (0.91 to 1.25) | 1.00 (0.84 to 1.18) |
|  | Weighted aHR (95% CI)^a^ | 1 [Reference] | 1.00 (0.89 to 1.13) | 0.92 (0.71 to 1.19) | 1.07 (0.91 to 1.26) | 0.97 (0.81 to 1.15) |
| *Asthma diagnosis only*^b^ | | | | | | |
|  | Cases, n (%) | 1,266 (1.2) | 113 (0.8) | 26 (1.0) | 52 (1.0) | 35 (0.6) |
|  | Unweighted HR (95% CI) | 1 [Reference] | 0.86 (0.71 to 1.05) | 1.02 (0.69 to 1.50) | 0.95 (0.72 to 1.26) | **0.69 (0.50 to 0.97)** |
|  | Weighted aHR (95% CI)^a^ | 1 [Reference] | 0.84 (0.69 to 1.03) | 0.95 (0.63 to 1.42) | 0.95 (0.71 to 1.26) | **0.68 (0.48 to 0.96)** |
| *Anaphylaxis* | | | | | | |
|  | Cases, n (%) | 955 (0.9) | 104 (0.8) | 28 (1.1) | 44 (0.9) | 32 (0.6) |
|  | Unweighted HR (95% CI) | 1 [Reference] | 0.94 (0.77 to 1.15) | 1.32 (0.90 to 1.92) | 1.00 (0.74 to 1.35) | 0.71 (0.50 to 1.01) |
|  | Weighted aHR (95% CI)^a^ | 1 [Reference] | 0.84 (0.68 to 1.04) | 1.14 (0.77 to 1.69) | 0.92 (0.67 to 1.27) | **0.62 (0.43 to 0.91)** |
| *Autoimmune disease* | | | | | | |
|  | Cases, n (%) | 137 (0.1) | 14 (0.1) | <5 | 6 (0.1) | 6 (0.1) |
|  | Unweighted HR (95% CI) | 1 [Reference] | 0.96 (0.55 to 1.67) | - | 1.00 (0.44 to 2.27) | 1.05 (0.46 to 2.37) |
|  | Weighted aHR (95% CI)^a^ | 1 [Reference] | 0.97 (0.55 to 1.71) | - | 1.04 (0.44 to 2.49) | 1.07 (0.47 to 2.47) |
| Abbreviations: CI, confidence interval; HR, unadjusted hazard ratio; aHR, adjusted hazard ratio; -, indeterminate (a stable estimate could not be generated due to the low number of outcomes).  All outcomes were identified from ICD-10-AM codes found in the principal and additional diagnosis fields of hospital inpatient records (**S1 Table**).  ^a^ Hazard ratios were weighted by inverse-probability of treatment factoring for maternal covariates including age, Aboriginal status, socioeconomic status, body mass index, parity, pre-existing medical conditions (asthma, essential hypertension, pre-existing diabetes), pregnancy complications (gestational diabetes, gestational hypertension, pre-eclampsia), smoking status during pregnancy, gestational age at first prenatal care visit, year and season of birth; models were additionally adjusted for child’s Aboriginal status.  ^b^ Sensitivity analysis restricting the definition of asthma to the presence of a diagnosis code of asthma alone (i.e., J45-J46). | | | | | | |
