# Supplementary material for: Prenatal influenza vaccination and allergic and autoimmune diseases in childhood: A longitudinal, population-based linked cohort study
Source: PLoS Med. 2022 Apr 5;19(4):e1003963. doi: 10.1371/journal.pmed.1003963 (PMC9017895; doi:10.1371/journal.pmed.1003963)
Supplement: S3 Table — (DOCX) [file pmed.1003963.s004.docx]

**S3 Table. Risk of allergic or autoimmune diseases associated with prenatal exposure to seasonal inactivated influenza vaccine among children <5 years of age who were born preterm (<37 weeks gestational age), by trimester of prenatal vaccination.**

|  | | **Unexposed to seasonal influenza vaccine during pregnancy**  **(N = 7,390)** | **Exposed to seasonal influenza vaccine during pregnancy**  **(N = 887)** | **Trimester of vaccine exposure** | | |
| --- | --- | --- | --- | --- | --- | --- |
|  |  |  |  | **First trimester**  **(N = 185)** | **Second trimester**  **(N = 441)** | **Third trimester**  **(N = 261)** |
| *Allergic or autoimmune disease* | | | | | | |
|  | Cases, n (%) | 700 (9.5) | 72 (8.1) | 16 (8.6) | 34 (7.7) | 22 (8.4) |
|  | Unweighted HR (95% CI) | 1 [Reference] | 0.90 (0.71 to 1.14) | 0.97 (0.59 to 1.59) | 0.82 (0.58 to 1.15) | 1.02 (0.67 to 1.55) |
|  | Weighted aHR (95% CI)^a^ | 1 [Reference] | 0.91 (0.70 to 1.17) | 0.99 (0.59 to 1.67) | 0.81 (0.56 to 1.16) | 1.03 (0.66 to 1.61) |
| *Allergic disease* | | | | | | |
|  | Cases, n (%) | 683 (9.2) | 70 (7.9) | 15 (8.1) | 34 (7.7) | 21 (8.0) |
|  | Unweighted HR (95% CI) | 1 [Reference] | 0.90 (0.70 to 1.15) | 0.92 (0.55 to 1.54) | 0.84 (0.59 to 1.18) | 1.00 (0.65 to 1.53) |
|  | Weighted aHR (95% CI)^a^ | 1 [Reference] | 0.91 (0.70 to 1.17) | 0.95 (0.56 to 1.63) | 0.83 (0.58 to 1.19) | 1.02 (0.65 to 1.61) |
| *Asthma diagnosis or wheezing* | | | | | | |
|  | Cases, n (%) | 398 (5.4) | 44 (5.0) | 8 (4.3) | 24 (5.4) | 12 (4.6) |
|  | Unweighted HR (95% CI) | 1 [Reference] | 0.98 (0.72 to 1.34) | 0.84 (0.42 to 1.68) | 1.02 (0.67 to 1.53) | 1.01 (0.57 to 1.79) |
|  | Weighted aHR (95% CI)^a^ | 1 [Reference] | 0.99 (0.71 to 1.38) | 0.89 (0.43 to 1.83) | 1.05 (0.68 to 1.62) | 0.96 (0.52 to 1.76) |
| *Asthma diagnosis only*^b^ | | | | | | |
|  | Cases, n (%) | 159 (2.2) | 18 (2.0) | <5 | 9 (2.0) | 5 (1.9) |
|  | Unweighted HR (95% CI) | 1 [Reference] | 1.06 (0.65 to 1.72) | - | 0.99 (0.51 to 1.94) | 1.13 (0.47 to 2.76) |
|  | Weighted aHR (95% CI)^a^ | 1 [Reference] | 1.12 (0.67 to 1.88) | - | 1.07 (0.53 to 2.16) | 1.10 (0.41 to 2.95) |
| *Anaphylaxis* | | | | | | |
|  | Cases, n (%) | 88 (1.2) | 10 (1.1) | <5 | <5 | <5 |
|  | Unweighted HR (95% CI) | 1 [Reference] | 1.04 (0.54 to 1.99) | - | - | - |
|  | Weighted aHR (95% CI)^a^ | 1 [Reference] | 1.01 (0.51 to 1.97) | - | - | - |
| *Autoimmune disease* | | | | | | |
|  | Cases, n (%) | 21 (0.3) | <5 | <5 | <5 | <5 |
|  | Unweighted HR (95% CI) | 1 [Reference] | - | - | - | - |
|  | Weighted aHR (95% CI)^a^ | 1 [Reference] | - | - | - | - |
| Abbreviations: CI, confidence interval; HR, unadjusted hazard ratio; aHR, adjusted hazard ratio; -, indeterminate (a stable estimate could not be generated due to the low number of outcomes).  All outcomes were identified from ICD-10-AM codes found in the principal and additional diagnosis fields of hospital inpatient records (**S1 Table**).  ^a^ Hazard ratios were weighted by inverse-probability of treatment factoring for maternal covariates including age, Aboriginal status, socioeconomic status, body mass index, parity, pre-existing medical conditions (asthma, essential hypertension, pre-existing diabetes), pregnancy complications (gestational diabetes, gestational hypertension, pre-eclampsia), smoking status during pregnancy, gestational age at first prenatal care visit, year and season of birth; models were additionally adjusted for child’s Aboriginal status.  ^b^ Sensitivity analysis restricting the definition of asthma to the presence of a diagnosis code of asthma alone (i.e., J45-J46). | | | | | | |
