# Supplementary material for: Prenatal influenza vaccination and allergic and autoimmune diseases in childhood: A longitudinal, population-based linked cohort study
Source: PLoS Med. 2022 Apr 5;19(4):e1003963. doi: 10.1371/journal.pmed.1003963 (PMC9017895; doi:10.1371/journal.pmed.1003963)
Supplement: S4 Table — (DOCX) [file pmed.1003963.s005.docx]

**S4 Table. Risk of inpatient admission (only) for allergic or autoimmune disease associated with prenatal exposure to seasonal inactivated influenza vaccine among children <5 years of age, by trimester of prenatal vaccination.**

|  | | **Unexposed to seasonal influenza vaccine during pregnancy**  **(N = 110,364)** | **Exposed to seasonal influenza vaccine during pregnancy**  **(N = 14,396)** | **Trimester of vaccine exposure** | | |
| --- | --- | --- | --- | --- | --- | --- |
|  |  |  |  | **First trimester**  **(N = 2,785)** | **Second trimester**  **(N = 5,558)** | **Third trimester**  **(N = 6,053)** |
| *Allergic or autoimmune disease* | | | | | | |
|  | Cases, n (%) | 3,431 (3.1) | 388 (2.7) | 71 (2.5) | 165 (3.0) | 152 (2.5) |
|  | Unweighted HR (95% CI) | 1 [Reference] | 0.99 (0.89 to 1.10) | 0.93 (0.73 to 1.17) | 1.01 (0.87 to 1.19) | 1.00 (0.85 to 1.18) |
|  | Weighted aHR (95% CI)^a^ | 1 [Reference] | 0.99 (0.89 to 1.11) | 0.93 (0.72 to 1.19) | 1.02 (0.87 to 1.21) | 0.99 (0.84 to 1.18) |
| *Allergic disease* | | | | | | |
|  | Cases, n (%) | 3,298 (3.0) | 375 (2.6) | 68 (2.4) | 161 (2.9) | 146 (2.4) |
|  | Unweighted HR (95% CI) | 1 [Reference] | 0.99 (0.89 to 1.11) | 0.92 (0.72 to 1.17) | 1.03 (0.88 to 1.20) | 1.00 (0.84 to 1.18) |
|  | Weighted aHR (95% CI)^a^ | 1 [Reference] | 0.99 (0.89 to 1.11) | 0.92 (0.71 to 1.20) | 1.04 (0.88 to 1.23) | 0.99 (0.83 to 1.18) |
| *Asthma diagnosis or wheezing* | | | | | | |
|  | Cases, n (%) | 2,759 (2.5) | 319 (2.2) | 57 (2.0) | 139 (2.5) | 123 (2.0) |
|  | Unweighted HR (95% CI) | 1 [Reference] | 1.02 (0.91 to 1.15) | 0.93 (0.71 to 1.21) | 1.06 (0.90 to 1.26) | 1.02 (0.85 to 1.22) |
|  | Weighted aHR (95% CI)^a^ | 1 [Reference] | 1.02 (0.90 to 1.15) | 0.95 (0.72 to 1.26) | 1.07 (0.89 to 1.28) | 1.00 (0.83 to 1.21) |
| *Asthma diagnosis only*^b^ | | | | | | |
|  | Cases, n (%) | 584 (0.5) | 53 (0.4) | 12 (0.4) | 26 (0.5) | 15 (0.2) |
|  | Unweighted HR (95% CI) | 1 [Reference] | 0.89 (0.67 to 1.18) | 1.03 (0.58 to 1.83) | 1.02 (0.69 to 1.52) | 0.67 (0.40 to 1.11) |
|  | Weighted aHR (95% CI)^a^ | 1 [Reference] | 0.87 (0.65 to 1.17) | 1.07 (0.58 to 1.96) | 1.03 (0.68 to 1.54) | 0.61 (0.36 to 1.05) |
| *Anaphylaxis* | | | | | | |
|  | Cases, n (%) | 268 (0.2) | 26 (0.2) | 9 (0.3) | 7 (0.1) | 10 (0.2) |
|  | Unweighted HR (95% CI) | 1 [Reference] | 0.82 (0.55 to 1.23) | 1.46 (0.75 to 2.84) | 0.55 (0.26 to 1.16) | 0.80 (0.42 to 1.50) |
|  | Weighted aHR (95% CI)^a^ | 1 [Reference] | 0.79 (0.52 to 1.21) | 1.59 (0.80 to 3.15) | 0.54 (0.24 to 1.20) | 0.67 (0.35 to 1.28) |
| *Autoimmune disease* | | | | | | |
|  | Cases, n (%) | 145 (0.1) | 15 (0.1) | <5 | 6 (0.1) | 6 (0.1) |
|  | Unweighted HR (95% CI) | 1 [Reference] | 0.97 (0.57 to 1.65) | - | 0.92 (0.41 to 2.09) | 1.01 (0.44 to 2.29) |
|  | Weighted aHR (95% CI)^a^ | 1 [Reference] | 0.97 (0.56 to 1.69) | - | 0.97 (0.41 to 2.31) | 1.04 (0.45 to 2.39) |
| Abbreviations: CI, confidence interval; HR, unadjusted hazard ratio; aHR, adjusted hazard ratio; -, indeterminate (a stable estimate could not be generated due to the low number of outcomes).  All outcomes were identified from ICD-10-AM codes found in the principal and additional diagnosis fields of hospital inpatient records (**S1 Table**).  ^a^ Hazard ratios were weighted by inverse-probability of treatment factoring for maternal covariates including age, Aboriginal status, socioeconomic status, body mass index, parity, pre-existing medical conditions (asthma, essential hypertension, pre-existing diabetes), pregnancy complications (gestational diabetes, gestational hypertension, pre-eclampsia), smoking status during pregnancy, gestational age at first prenatal care visit, year and season of birth; models were additionally adjusted for child’s Aboriginal status.  ^b^ Sensitivity analysis restricting the definition of asthma to the presence of a diagnosis code of asthma alone (i.e., J45-J46). | | | | | | |
