# Supplementary material for: Prenatal influenza vaccination and allergic and autoimmune diseases in childhood: A longitudinal, population-based linked cohort study
Source: PLoS Med. 2022 Apr 5;19(4):e1003963. doi: 10.1371/journal.pmed.1003963 (PMC9017895; doi:10.1371/journal.pmed.1003963)
Supplement: S5 Table — (DOCX) [file pmed.1003963.s006.docx]

**S5 Table. Risk of allergic or autoimmune diseases associated with prenatal exposure to seasonal inactivated influenza vaccine among children between 6 months and <5 years of age, by trimester of prenatal vaccination.**

|  | | **Unexposed to seasonal influenza vaccine during pregnancy**  **(N = 110,158)** | **Exposed to seasonal influenza vaccine during pregnancy**  **(N = 14,373)** | **Trimester of vaccine exposure** | | |
| --- | --- | --- | --- | --- | --- | --- |
|  |  |  |  | **First trimester**  **(N = 2,778)** | **Second trimester**  **(N = 5,551)** | **Third trimester**  **(N = 6,044)** |
| *Allergic or autoimmune disease* | | | | | | |
|  | Cases, n (%) | 6,898 (6.3) | 811 (5.6) | 155 (5.6) | 350 (6.3) | 306 (5.1) |
|  | Unweighted HR (95% CI) | 1 [Reference] | 1.04 (0.96 to 1.11) | 1.02 (0.87 to 1.19) | 1.08 (0.97 to 1.20) | 1.00 (0.89 to 1.12) |
|  | Weighted aHR (95% CI)^a^ | 1 [Reference] | 1.02 (0.94 to 1.10) | 1.00 (0.84 to 1.18) | 1.07 (0.95 to 1.19) | 0.98 (0.87 to 1.10) |
| *Allergic disease* | | | | | | |
|  | Cases, n (%) | 6,771 (6.1) | 799 (5.6) | 152 (5.5) | 348 (6.3) | 299 (4.9) |
|  | Unweighted HR (95% CI) | 1 [Reference] | 1.04 (0.96 to 1.12) | 1.01 (0.86 to 1.19) | 1.09 (0.98 to 1.22) | 0.99 (0.88 to 1.11) |
|  | Weighted aHR (95% CI)^a^ | 1 [Reference] | 1.02 (0.94 to 1.10) | 1.00 (0.84 to 1.18) | 1.08 (0.96 to 1.21) | 0.97 (0.86 to 1.10) |
| *Asthma diagnosis or wheezing* | | | | | | |
|  | Cases, n (%) | 3,325 (3) | 379 (2.6) | 68 (2.4) | 167 (3.0) | 144 (2.4) |
|  | Unweighted HR (95% CI) | 1 [Reference] | 1.02 (0.92 to 1.14) | 0.93 (0.73 to 1.19) | 1.07 (0.92 to 1.25) | 1.01 (0.85 to 1.19) |
|  | Weighted aHR (95% CI)^a^ | 1 [Reference] | 1.01 (0.90 to 1.13) | 0.93 (0.72 to 1.21) | 1.07 (0.91 to 1.26) | 0.98 (0.82 to 1.17) |
| *Asthma diagnosis only*^b^ | | | | | | |
|  | Cases, n (%) | 1,412 (1.3) | 131 (0.9) | 30 (1.1) | 61 (1.1) | 40 (0.7) |
|  | Unweighted HR (95% CI) | 1 [Reference] | 0.90 (0.75 to 1.07) | 1.05 (0.73 to 1.50) | 0.98 (0.76 to 1.27) | 0.73 (0.53 to 1.00) |
|  | Weighted aHR (95% CI)^a^ | 1 [Reference] | 0.88 (0.73 to 1.07) | 1.00 (0.68 to 1.46) | 0.99 (0.75 to 1.29) | **0.71 (0.51 to 0.99)** |
| *Anaphylaxis* | | | | | | |
|  | Cases, n (%) | 880 (0.8) | 96 (0.7) | 25 (0.9) | 39 (0.7) | 32 (0.5) |
|  | Unweighted HR (95% CI) | 1 [Reference] | 0.97 (0.79 to 1.20) | 1.31 (0.88 to 1.95) | 0.96 (0.70 to 1.33) | 0.82 (0.58 to 1.17) |
|  | Weighted aHR (95% CI)^a^ | 1 [Reference] | 0.84 (0.68 to 1.05) | 1.15 (0.76 to 1.74) | 0.86 (0.62 to 1.21) | **0.68 (0.47 to 0.99)** |
| *Autoimmune disease* | | | | | | |
|  | Cases, n (%) | 144 (0.1) | 14 (0.1) | <5 | <5 | 7 (0.1) |
|  | Unweighted HR (95% CI) | 1 [Reference] | 0.93 (0.54 to 1.61) | - | - | 1.22 (0.57 to 2.61) |
|  | Weighted aHR (95% CI)^a^ | 1 [Reference] | 0.91 (0.52 to 1.61) | - | - | 1.17 (0.54 to 2.55) |
| Abbreviations: CI, confidence interval; HR, unadjusted hazard ratio; aHR, adjusted hazard ratio; -, indeterminate (a stable estimate could not be generated due to the low number of outcomes).  All outcomes were identified from ICD-10-AM codes found in the principal and additional diagnosis fields of hospital inpatient and emergency department presentation records, and from the presenting symptom code found in the emergency department presentation records (**S1 Table**).  ^a^ Hazard ratios were weighted by inverse-probability of treatment factoring for maternal covariates including age, Aboriginal status, socioeconomic status, body mass index, parity, pre-existing medical conditions (asthma, essential hypertension, pre-existing diabetes), pregnancy complications (gestational diabetes, gestational hypertension, pre-eclampsia), smoking status during pregnancy, gestational age at first prenatal care visit, year and season of birth; models were additionally adjusted for child’s Aboriginal status.  ^b^ Sensitivity analysis restricting the definition of asthma to the presence of a diagnosis code of asthma alone (i.e., J45-J46). | | | | | | |
