# Supplementary material for: Prenatal influenza vaccination and allergic and autoimmune diseases in childhood: A longitudinal, population-based linked cohort study
Source: PLoS Med. 2022 Apr 5;19(4):e1003963. doi: 10.1371/journal.pmed.1003963 (PMC9017895; doi:10.1371/journal.pmed.1003963)
Supplement: S6 Table — (DOCX) [file pmed.1003963.s007.docx]

**S6 Table. Risk of allergic or autoimmune diseases associated with prenatal exposure to seasonal inactivated influenza vaccine among one randomly selected child per mother, by trimester of prenatal vaccination.**

|  | | **Unexposed to seasonal influenza vaccine during pregnancy**  **(N = 93,799)** | **Exposed to seasonal influenza vaccine during pregnancy**  **(N = 12,407)** | **Trimester of vaccine exposure** | | |
| --- | --- | --- | --- | --- | --- | --- |
|  |  |  |  | **First trimester**  **(N = 2,397)** | **Second trimester**  **(N = 4,798)** | **Third trimester**  **(N = 5,212)** |
| *Allergic or autoimmune disease* | | | | | | |
|  | Cases, n (%) | 6,512 (6.9) | 800 (6.4) | 156 (6.5) | 341 (7.1) | 303 (5.8) |
|  | Unweighted HR (95% CI) | 1 [Reference] | 1.05 (0.97 to 1.13) | 1.05 (0.90 to 1.23) | 1.09 (0.98 to 1.22) | 1.00 (0.89 to 1.13) |
|  | Weighted aHR (95% CI)^a^ | 1 [Reference] | 1.03 (0.95 to 1.11) | 1.02 (0.86 to 1.21) | 1.07 (0.95 to 1.20) | 1.00 (0.88 to 1.13) |
| *Allergic disease* | | | | | | |
|  | Cases, n (%) | 6,395 (6.8) | 789 (6.4) | 154 (6.4) | 337 (7.0) | 298 (5.7) |
|  | Unweighted HR (95% CI) | 1 [Reference] | 1.05 (0.98 to 1.13) | 1.05 (0.90 to 1.24) | 1.10 (0.98 to 1.22) | 1.00 (0.89 to 1.13) |
|  | Weighted aHR (95% CI)^a^ | 1 [Reference] | 1.03 (0.96 to 1.12) | 1.03 (0.87 to 1.22) | 1.07 (0.96 to 1.21) | 1.00 (0.88 to 1.13) |
| *Asthma diagnosis or wheezing* | | | | | | |
|  | Cases, n (%) | 2,892 (3.1) | 334 (2.7) | 64 (2.7) | 140 (2.9) | 130 (2.5) |
|  | Unweighted HR (95% CI) | 1 [Reference] | 1.02 (0.91 to 1.14) | 0.99 (0.77 to 1.27) | 1.02 (0.86 to 1.20) | 1.03 (0.86 to 1.23) |
|  | Weighted aHR (95% CI)^a^ | 1 [Reference] | 1.01 (0.89 to 1.13) | 0.98 (0.75 to 1.28) | 1.02 (0.85 to 1.22) | 1.01 (0.84 to 1.22) |
| *Asthma diagnosis only*^b^ | | | | | | |
|  | Cases, n (%) | 1,225 (1.3) | 116 (0.9) | 28 (1.2) | 51 (1.1) | 37 (0.7) |
|  | Unweighted HR (95% CI) | 1 [Reference] | 0.90 (0.74 to 1.09) | 1.10 (0.76 to 1.6) | 0.93 (0.70 to 1.23) | 0.76 (0.55 to 1.06) |
|  | Weighted aHR (95% CI)^a^ | 1 [Reference] | 0.88 (0.72 to 1.07) | 1.01 (0.68 to 1.49) | 0.93 (0.69 to 1.25) | 0.74 (0.53 to 1.05) |
| *Anaphylaxis* | | | | | | |
|  | Cases, n (%) | 862 (0.9) | 100 (0.8) | 26 (1.1) | 43 (0.9) | 31 (0.6) |
|  | Unweighted HR (95% CI) | 1 [Reference] | 1.00 (0.81 to 1.22) | 1.33 (0.90 to 1.97) | 1.05 (0.78 to 1.43) | 0.77 (0.54 to 1.11) |
|  | Weighted aHR (95% CI)^a^ | 1 [Reference] | 0.89 (0.72 to 1.11) | 1.19 (0.79 to 1.79) | 0.96 (0.69 to 1.32) | 0.68 (0.47 to 1.00) |
| *Autoimmune disease* | | | | | | |
|  | Cases, n (%) | 136 (0.1) | 13 (0.1) | <5 | 6 (0.1) | 5 (0.1) |
|  | Unweighted HR (95% CI) | 1 [Reference] | 0.88 (0.50 to 1.56) | - | 0.97 (0.43 to 2.20) | 0.88 (0.36 to 2.15) |
|  | Weighted aHR (95% CI)^a^ | 1 [Reference] | 0.86 (0.47 to 1.56) | - | 1.02 (0.43 to 2.45) | 0.84 (0.33 to 2.14) |
| Abbreviations: CI, confidence interval; HR, crude hazard ratio; aHR, adjusted hazard ratio; -, indeterminate (a stable estimate could not be generated due to the low number of outcomes).  All outcomes were identified from ICD-10-AM codes found in the principal and additional diagnosis fields of hospital inpatient records (**S1 Table**).  ^a^ Hazard ratios were weighted by inverse-probability of treatment factoring for maternal covariates including age, Aboriginal status, socioeconomic status, body mass index, parity, pre-existing medical conditions (asthma, essential hypertension, pre-existing diabetes), pregnancy complications (gestational diabetes, gestational hypertension, pre-eclampsia), smoking status during pregnancy, gestational age at first prenatal care visit, year and season of birth; models were additionally adjusted for child’s Aboriginal status.  ^b^ Sensitivity analysis restricting the definition of asthma to the presence of a diagnosis code of asthma alone (i.e., J45-J46). | | | | | | |
