# Supplementary material for: Prenatal influenza vaccination and allergic and autoimmune diseases in childhood: A longitudinal, population-based linked cohort study
Source: PLoS Med. 2022 Apr 5;19(4):e1003963. doi: 10.1371/journal.pmed.1003963 (PMC9017895; doi:10.1371/journal.pmed.1003963)
Supplement: S7 Table — (DOCX) [file pmed.1003963.s008.docx]

**S7 Table. Risk of allergic or autoimmune diseases associated with prenatal exposure to seasonal inactivated influenza vaccine among children <5 years of age matched by year and month of birth, by trimester of prenatal vaccination.**

|  | | **Unexposed to seasonal influenza vaccine during pregnancy**  **(N = 110,364)** | **Exposed to seasonal influenza vaccine during pregnancy**  **(N = 14,396)** | **Trimester of vaccine exposure** | | |
| --- | --- | --- | --- | --- | --- | --- |
|  |  |  |  | **First trimester**  **(N = 2,785)** | **Second trimester**  **(N = 5,558)** | **Third trimester**  **(N = 6,053)** |
| *Allergic or autoimmune disease* | | | | | | |
|  | Cases, n (%) | 7,655 (6.9) | 913 (6.3) | 171 (6.1) | 395 (7.1) | 347 (5.7) |
|  | Unweighted HR (95% CI) | 1 [Reference] | 1.04 (0.97 to 1.12) | 0.96 (0.83 to 1.12) | 1.11 (1.00 to 1.23) | 1.01 (0.91 to 1.13) |
|  | Weighted aHR (95% CI)^a^ | 1 [Reference] | 1.03 (0.96 to 1.11) | 0.97 (0.83 to 1.14) | 1.10 (0.99 to 1.22) | 1.00 (0.89 to 1.13) |
| *Allergic disease* | | | | | | |
|  | Cases, n (%) | 7,518 (6.8) | 899 (6.2) | 168 (6.0) | 391 (7.0) | 340 (5.6) |
|  | Unweighted HR (95% CI) | 1 [Reference] | 1.04 (0.97 to 1.12) | 0.96 (0.82 to 1.12) | 1.12 (1.01 to 1.24) | 1.01 (0.90 to 1.13) |
|  | Weighted aHR (95% CI)^a^ | 1 [Reference] | 1.03 (0.96 to 1.11) | 0.96 (0.82 to 1.13) | 1.11 (1.00 to 1.23) | 1.00 (0.89 to 1.13) |
| *Asthma diagnosis or wheezing* | | | | | | |
|  | Cases, n (%) | 3,375 (3.1) | 382 (2.7) | 68 (2.4) | 169 (3.0) | 145 (2.4) |
|  | Unweighted HR (95% CI) | 1 [Reference] | 0.92 (0.77 to 1.11) | 1.07 (0.74 to 1.54) | 1.02 (0.79 to 1.33) | 0.74 (0.53 to 1.03) |
|  | Weighted aHR (95% CI)^a^ | 1 [Reference] | 0.93 (0.77 to 1.13) | 1.13 (0.77 to 1.66) | 0.98 (0.76 to 1.28) | 0.76 (0.54 to 1.07) |
| *Asthma diagnosis only*^b^ | | | | | | |
|  | Cases, n (%) | 1,425 (1.3) | 131 (0.9) | 30 (1.1) | 61 (1.1) | 40 (0.7) |
|  | Unweighted HR (95% CI) | 1 [Reference] | 1.03 (0.93 to 1.15) | 0.89 (0.70 to 1.13) | 1.11 (0.95 to 1.30) | 1.04 (0.87 to 1.24) |
|  | Weighted aHR (95% CI)^a^ | 1 [Reference] | 1.03 (0.92 to 1.15) | 0.92 (0.72 to 1.18) | 1.11 (0.95 to 1.31) | 1.00 (0.84 to 1.20) |
| *Anaphylaxis* | | | | | | |
|  | Cases, n (%) | 1,043 (0.9) | 114 (0.8) | 30 (1.1) | 48 (0.9) | 36 (0.6) |
|  | Unweighted HR (95% CI) | 1 [Reference] | 0.90 (0.74 to 1.10) | 1.18 (0.82 to 1.71) | 0.92 (0.69 to 1.24) | 0.72 (0.51 to 1.01) |
|  | Weighted aHR (95% CI)^a^ | 1 [Reference] | 0.89 (0.73 to 1.10) | 1.17 (0.81 to 1.70) | 0.90 (0.67 to 1.22) | **0.69 (0.49 to 0.97)** |
| *Autoimmune disease* | | | | | | |
|  | Cases, n (%) | 158 (0.1) | 16 (0.1) | <5 | 6 (0.1) | 7 (0.1) |
|  | Unweighted HR (95% CI) | 1 [Reference] | 0.99 (0.58 to 1.68) | - | 0.82 (0.35 to 1.90) | 1.16 (0.53 to 2.55) |
|  | Weighted aHR (95% CI)^a^ | 1 [Reference] | 0.96 (0.57 to 1.64) | - | 0.83 (0.34 to 2.02) | 1.08 (0.51 to 2.29) |
| Abbreviations: CI, confidence interval; HR, unadjusted hazard ratio; aHR, adjusted hazard ratio; -, indeterminate (a stable estimate could not be generated due to the low number of outcomes).  All outcomes were identified from ICD-10-AM codes found in the principal and additional diagnosis fields of hospital inpatient and emergency department presentation records, and from the presenting symptom code found in the emergency department presentation records (**S1 Table**).  ^a^ Hazard ratios were weighted by inverse-probability of treatment factoring for maternal covariates including age, Aboriginal status, socioeconomic status, body mass index, parity, pre-existing medical conditions (asthma, essential hypertension, pre-existing diabetes), pregnancy complications (gestational diabetes, gestational hypertension, pre-eclampsia), smoking status during pregnancy, gestational age at first prenatal care visit, models were additionally adjusted for child’s Aboriginal status.  ^b^ Sensitivity analysis restricting the definition of asthma to the presence of a diagnosis code of asthma alone (i.e., J45-J46). | | | | | | |
