# Supplementary material for: Prenatal influenza vaccination and allergic and autoimmune diseases in childhood: A longitudinal, population-based linked cohort study
Source: PLoS Med. 2022 Apr 5;19(4):e1003963. doi: 10.1371/journal.pmed.1003963 (PMC9017895; doi:10.1371/journal.pmed.1003963)
Supplement: S8 Table — (DOCX) [file pmed.1003963.s009.docx]

**S8 Table. Risk of all-cause injury associated with prenatal exposure to seasonal inactivated influenza vaccination among children <5 years of age, by trimester of prenatal vaccination.**

|  | | **Unexposed to seasonal influenza vaccine during pregnancy**  **(N = 110,364)** | **Exposed to seasonal influenza vaccine during pregnancy**  **(N = 14,396)** | **Trimester of vaccine exposure** | | |
| --- | --- | --- | --- | --- | --- | --- |
|  |  |  |  | **First trimester**  **(N = 2,785)** | **Second trimester**  **(N = 5,558)** | **Third trimester**  **(N = 6,053)** |
| *Hospital inpatient admissions and emergency department presentations* | | | | | | |
|  | Cases, n (%) | 19,452 (17.6) | 2,278 (15.8) | 446 (16.0) | 966 (17.4) | 866 (14.3) |
|  | Unweighted HR (95% CI) | 1 [Reference] | **1.06 (1.02 to 1.11)** | 1.07 (0.98 to 1.18) | **1.08 (1.01 to 1.15)** | 1.04 (0.97 to 1.11) |
|  | Weighted aHR (95% CI)^a^ | 1 [Reference] | 1.04 (0.99 to 1.11) | 1.05 (0.95 to 1.16) | 1.05 (0.99 to 1.13) | 1.02 (0.94 to 1.09) |
| *Hospital inpatient admissions only* | | | | | | |
|  | Cases, n (%) | 3,173 (2.9) | 355 (2.5) | 74 (2.7) | 138 (2.5) | 143 (2.4) |
|  | Unweighted HR (95% CI) | 1 [Reference] | 1.02 (0.92 to 1.14) | 1.10 (0.88 to 1.39) | 0.95 (0.80 to 1.13) | 1.06 (0.90 to 1.25) |
|  | Weighted aHR (95% CI)^a^ | 1 [Reference] | 1.01 (0.90 to 1.14) | 1.11 (0.87 to 1.41) | 0.93 (0.78 to 1.12) | 1.05 (0.87 to 1.27) |
| Abbreviations: CI, confidence interval; HR, unadjusted hazard ratio; aHR, adjusted hazard ratio; -, indeterminate (a stable estimate could not be generated due to the low number of outcomes).  All-cause injury was identified from the ICD-10-AM codes: S01-S09, S11-S19, S21-S29, S31-S39, S41-S49, S51-S59, S61-S69, S71-S79, S81-S89, S91-S99, found in the principal and additional diagnosis fields of hospital inpatient records and/or emergency department presentation records, and from the presenting symptom code found in the emergency department presentation records (**S1 Table**).  ^a^ Hazard ratios were weighted by inverse-probability of treatment factoring for maternal covariates including age, Aboriginal status, socioeconomic status, body mass index, parity, pre-existing medical conditions (asthma, essential hypertension, pre-existing diabetes), pregnancy complications (gestational diabetes, gestational hypertension, pre-eclampsia), smoking status during pregnancy, gestational age at first prenatal care visit, year and season of birth; models were additionally adjusted for child’s Aboriginal status. | | | | | | |
