# Supplementary figures and images for: Prenatal influenza vaccination and allergic and autoimmune diseases in childhood: A longitudinal, population-based linked cohort study
Source: PLoS Med. 2022 Apr 5;19(4):e1003963. doi: 10.1371/journal.pmed.1003963 (PMC9017895; doi:10.1371/journal.pmed.1003963)

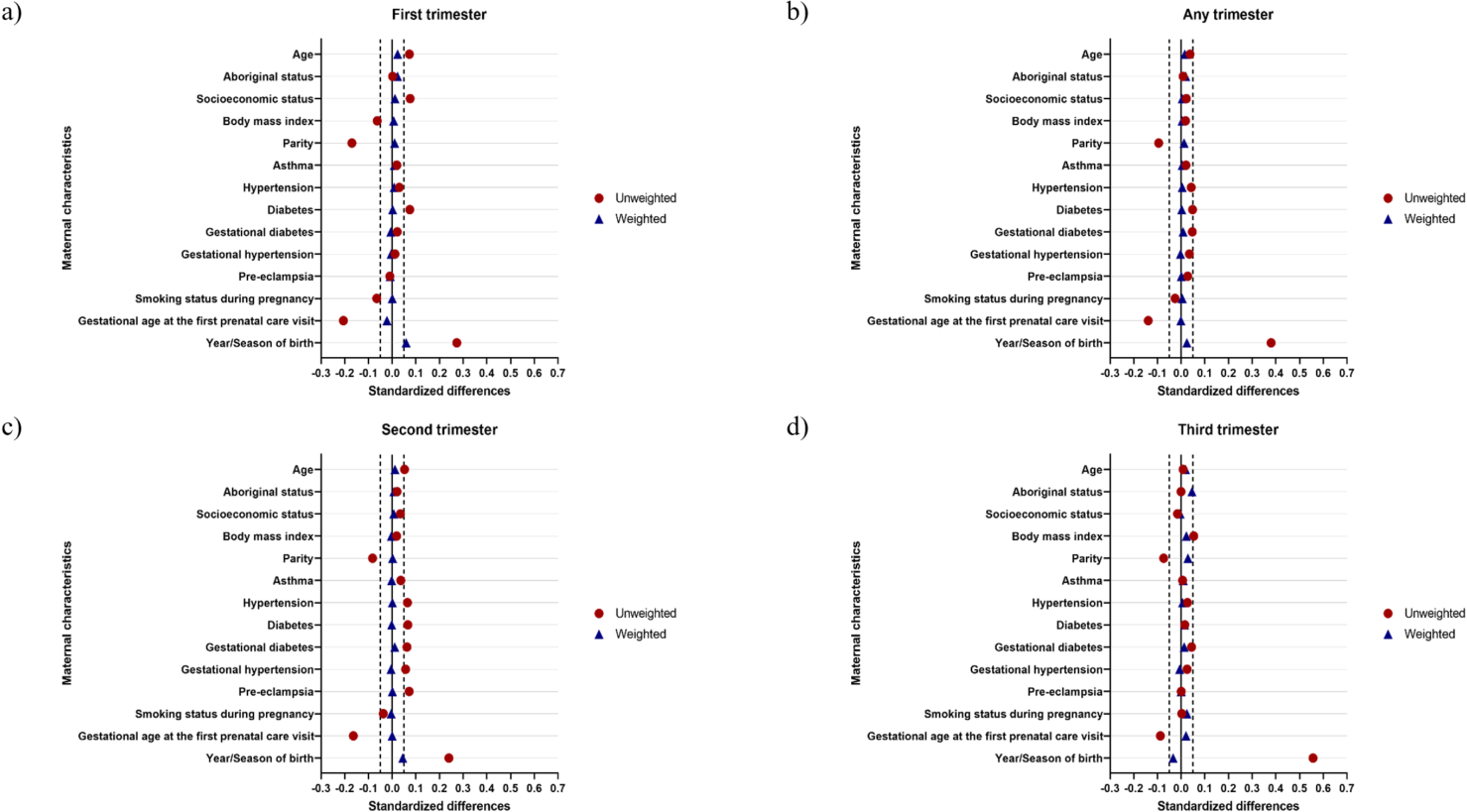

Supplement: S1 Fig — Balance of standardized differences of maternal covariates before and after inverse probability treatment weighting, by trimester of vaccination: (a) maternally vaccinated during any trimester; (b) maternally vaccinated during the first trimester; (c) maternally vaccinated during the second trimester; or (d) maternally vaccinated during the third trimester. (TIF) [file pmed.1003963.s010.tif]
