# Supplementary material for: Prenatal influenza vaccination and allergic and autoimmune diseases in childhood: A longitudinal, population-based linked cohort study
Source: PLoS Med. 2022 Apr 5;19(4):e1003963. doi: 10.1371/journal.pmed.1003963 (PMC9017895; doi:10.1371/journal.pmed.1003963)
Supplement: S1 Appendix — (DOCX) [file pmed.1003963.s011.docx]

**S1 Appendix. Sample STATA code for running unweighted/unadjusted and weighted/adjusted models to estimate the association between prenatal exposure to seasonal inactivated influenza vaccine and allergic and/or autoimmune diseases in children.**

********************************************************

SAMPLE CODE DESCRIPTION

********************************************************

This example code summarizes the procedures used to generate the inverse-probability of treatment weights and unweighted and weighted to estimate the associations between maternal influenza vaccination and pediatric health outcomes.

Required Variables:

Cohort:

1. child = Child’s ID

2. mother = Mother’s ID

Exposure:

1. fluvaccyn (0 = Child is maternally unvaccinated; 1 = Child is maternally vaccinated)

2. fluvacctri (1 = Maternally vaccinated during first trimester; 2 = Maternally vaccinated during second trimester; 3 = Maternally vaccinated during third trimester)

Outcomes (0 = No; 1 = Yes):

1. allergy_autoimmune = Allergic or autoimmune disease

2. allergy = Allergic disease

3. asthma1 = Asthma diagnosis only

4. asthma2 = Asthma or wheezing diagnosis

5. anaphylaxis = Anaphylaxis

6. autoimmune disease = Autoimmune disease

Covariates:

1. matage = Maternal age

2. bmi = Body mass index

3. mother_aboriginal_status = Mother's Aboriginal and/or Torres Strait Islander status (0 = Non-Aboriginal; 1 = Aboriginal and/or Torres Strait Islander)

4. parity = Parity

5. prenat_care_trimester = Trimester of first prenatal care visit

6. year_season_birth = Year and season of birth (e.g., 2012 Summer, 2013 Autumn, 2014 Winter, 2015 Spring, etc...)

7. smoke = Maternal smoking status during pregnancy (0 = No; 1 = Yes)

8. seifa5 = Socioeconomic status (1 = Quintile 1 [most disadvantaged] through to 5 = Quintile 5 [Least disadvantaged])

9. asthma_mum = Maternal history of asthma (0 = No; 1 = Yes)

10. hypertension = Maternal history of hypertension (0 = No; 1 = Yes)

11. diabetes = Maternal history of diabetes mellitus (0 = No; 1 = Yes)

12. gestdiabetes = Diagnosis of gestational diabetes during pregnancy for specific child (0 = No; 1 = Yes)

13. gesthypertension = Diagnosis of gestational hypertension during pregnancy for specific child (0 = No; 1 = Yes)

14. preeclampsia = Diagnosis of pre-eclampsia during pregnancy for specific child (0 = No; 1 = Yes)

15. child_aboriginal_status = Child's Aboriginal and/or Torres Strait Islander status (0 = Non-Aboriginal; 1 = Aboriginal and/or Torres Strait Islander)

Person-time variables:

1.1. origin = Child's date of birth or date when child is 6 months of age (sensitivity analysis)

2.1. start = Child's date of birth or date when child is 6 months of age (sensitivity analysis)

3. end = Earliest of: a) the date the child reached 5 years of age,

b) the last date of available data provided by the WA Data Linkage Branch (i.e., 1 July 2017),

c) the date the child died, or

d) the date of the event.

*********************************************************************

BEGINNING OF SAMPLE CODE

*********************************************************************

Apply propensity scores/inverse-probability treatment weights (IPTW): Probability of maternal vaccination

logistic fluvaccyn matage bmi i.mother_aboriginal_status parity i.prenat_care_trimester year_season_birth i.smoke i.seifa5 i.asthma_mum i.hypertension i.diabetes i.gestdiabetes i.gesthypertension i.preeclampsia

predict p

gen IPTW=1

replace IPTW=1/p if fluvaccyn==1

replace IPTW=1/(1-p) if fluvaccyn==0

**************************************************

Restrictions for sensitivity analysis for one randomly selected child per mother:

sort rootnum bdob, stable

by mother: gen morbseq=_n

tab morbseq // Max: 4 children per mother

sort mother random

drop if mother==mother[_n-1] // Restricted to 1 randomly selected child per mother

**************************************************

Stratified analysis by trimester of vaccination and preterm birth status:

Trimester of vaccination:

1. keep if fluvaccyn==0 | fluvacctri==1 // (compare 1st trimester vaccinated children with maternally unvaccinated children)

2. keep if fluvaccyn==0 | fluvacctri==2 // (compare 2nd trimester vaccinated children with maternally unvaccinated children)

3. keep if fluvaccyn==0 | fluvacctri==3 // (compare 3rd trimester vaccinated children with maternally unvaccinated children)

Preterm birth status (0 = No; 1 = Yes):

1. keep if preterm==0 // (restrict to term children)

2. keep if preterm==1 // (restrict to preterm children)

**************************************************

Perform unweighted/unadjusted analysis using Cox proportional hazards regression models

stset end, origin(origin) enter(start) exit(time .) fail(allergy_autoimmune=1) id(child) scale(365.25)

stcox i.fluvaccyn, vce(robust) // Generates robust effect estimate

stset, clear

Perform unweighted/unadjusted analysis using Cox proportional hazards regression models and weighted by propensity scores (i.e., IPTW)

stset end [pweight=IPTW], origin(origin) enter(start) exit(time .) fail(allergy_autoimmune=1) id(child) scale(365.25)

stcox i.fluvaccyn i.child_aboriginal_status, vce(robust) // Generates robust effect estimate adjusted by child's Aboriginal and/or Torres Strait Islander status

stset, clear
