# Supplementary material for: Prenatal influenza vaccination and allergic and autoimmune diseases in childhood: A longitudinal, population-based linked cohort study
Source: PLoS Med. 2022 Apr 5;19(4):e1003963. doi: 10.1371/journal.pmed.1003963 (PMC9017895; doi:10.1371/journal.pmed.1003963)
Supplement: Attachment — Submitted filename: PLoS Med_Foo_Response Letter and Form.docx [file pmed.1003963.s012.docx]

Dr Raffaella Bosurgi

Editor-in-Chief, *PLoS Medicine*

Carlyle House

Carlyle Road

Cambridge, CB4 3DN

United Kingdom

Dear Dr Bosurgi, reviewers & members of the Editorial Board at *PLoS Medicine*,

Thank you for your recent review of our paper, “Prenatal influenza vaccination and allergic and autoimmune diseases in childhood: A longitudinal, population-based cohort study” (Manuscript ID: PMEDICINE-D-21-02861R1). We have found the Academic Editor’s comments helpful in revising and strengthening our manuscript.

To facilitate further review, we have provided a point-by-point response to each editor and reviewer comment. We have also provided a revised manuscript with changes indicated in “track changes”. We are hopeful that you will find this revised manuscript acceptable for publication in *PLoS Medicine*.

Sincerely,

Damien Foo, on behalf of all authors

PhD Candidate

Curtin School of Population Health

Curtin University

**PMEDICINE-D-21-02861R1 – Prenatal influenza vaccination and allergic and autoimmune diseases in childhood: A longitudinal, population-based cohort study – by Damien Foo et al.**

| **Editor/Reviewer Comments** | **Author’s Response** | **Reference page** | **Change  Approved?**  **FOR**  **EDITORIAL USE ONLY** |
| --- | --- | --- | --- |
| **RESPONSE TO THE ACADEMIC EDITOR** | | |  |
| 1) The authors found a protective association between immunization in third trimester and asthma and anaphylaxis. My concern was that there could be a risk of residual confounding. The reason is that in most settings there is an association between season at birth and allergic disorders. Influenza vaccination has also a seasonal variation. Third trimester vaccination implies that birth happens soon after vaccination - in the same season or the next. The authors could easily show whether there is a risk of residual confounding - by reporting whether asthma and anaphylaxis are associated with season of birth or not. | 1) Thank you for these comments. While we acknowledge the association between season of birth and allergic disorders. We did not observe strong seasonality in these conditions in our study cohort in our setting (please see the below figures).  Allergic or autoimmune disease:    Allergic disease:    Asthma diagnosis only:    Asthma (or wheezing):    Anaphylaxis:    However, we agree with the Editor that seasonality is a potential confounder of particular concern when considering exposure to seasonal influenza vaccines. For this reason, we included year and season of birth in our propensity score/inverse-probability of treatment weights analyses. From S1 Figure, we were able to confirm that our cohort was balanced based on season of birth. However, we acknowledge the persisting concern raised by the Editor and have conducted an additional sensitivity analysis as requested, where we match the cohort based on year and month of birth (S7 Table). |  |  |
| 2) I observe another problem that easily could be fixed: the association between third trimester vaccination and asthma and anaphylaxis is presented as "a strong protective association" in author summary bullets. In Discussion the authors write: "We observed a negative association of anaphylaxis and some indications of low risk of asthma following seasonal IIV given during the third trimester." I believe the author summary bullet should be modified in line with the more careful extression they have in Discussion. | 2) Thank you for this suggestion. The Author Summary has been modified in line with the Discussion section.  “We identified a negative association between anaphylaxis and influenza vaccination during the third trimester”. | Author Summary |  |
| Requests from the editors:   3) We suggest conducting a sensitivity analysis to assess the potential impact of season at birth on the associations you have observed in your study. This will be subject to statistical re-review. | 3) As described in our response to the Editor, Q1 above, we have performed an additional sensitivity analysis where we match maternally vaccinated and unvaccinated children by calendar year and month of birth and removed year and season of birth from the propensity score weights.  Results from this analysis (now described in S7 Table), show that although we no longer observe associations with asthma, however, associations between anaphylaxis and third trimester vaccination persist (weighted aHR: 0.69 [95% CI: 0.49 to 0.97]).  Thank you for this suggestion. | Methods: Statistical analyses (Paragraph 3); Results: Asthma; Results: Anaphylaxis; S7 Table |  |
| **RESPONSE TO REVIEWER 1** | | |  |
| 1) Thanks authors for their effort to improve the manuscript. I am satisfied with the response and revision. No further issues needing attention. | 1) We thank Reviewer 1 for their helpful comments and suggestions. |  |  |
