# Supplementary material for: Prenatal influenza vaccination and allergic and autoimmune diseases in childhood: A longitudinal, population-based linked cohort study
Source: PLoS Med. 2022 Apr 5;19(4):e1003963. doi: 10.1371/journal.pmed.1003963 (PMC9017895; doi:10.1371/journal.pmed.1003963)
Supplement: Attachment — Submitted filename: PLoS Med_Foo_Response Letter and Form.docx [file pmed.1003963.s013.docx]

Dr Raffaella Bosurgi

Editor-in-Chief, *PLoS Medicine*

Carlyle House

Carlyle Road

Cambridge, CB4 3DN

United Kingdom

Dear Dr Bosurgi, reviewers & members of the Editorial Board at *PLoS Medicine*,

Thank you for your recent review of our paper, “Prenatal influenza vaccination and allergic and autoimmune diseases in childhood: A longitudinal, population-based linked cohort study” (Manuscript ID: PMEDICINE-D-21-02861R1). We have found the Academic Editor’s comments helpful in revising and strengthening our manuscript.

To facilitate further review, we have provided a point-by-point response to each editor and reviewer comment. We have also provided a revised manuscript with changes indicated in “track changes”. We are hopeful that you will find this revised manuscript acceptable for publication in *PLoS Medicine*.

Sincerely,

Damien Foo, on behalf of all authors

PhD Candidate

Curtin School of Population Health

Curtin University

**PMEDICINE-D-21-02861R1 – Prenatal influenza vaccination and allergic and autoimmune diseases in childhood: A longitudinal, population-based linked cohort study – by Damien Foo et al.**

| **Editor/Reviewer Comments** | **Author’s Response** | **Reference page** | **Change  Approved?**  **FOR**  **EDITORIAL USE ONLY** |
| --- | --- | --- | --- |
| **RESPONSE TO THE EDITORS** | | |  |
| Data availability:   1) Please note that a study author cannot be a contact person for programming code. Please provide an alternative appropriate contact (web or email address) for inquiries, or consider publishing your code in GitHub (please provide a link). | We have attached the key code for the primary and secondary analyses to the Supplement as “S1 Appendix”. | S1 Appendix |  |
| Abstract Methods and Findings:   2) Please define CI at first use | Thank you for the suggestion. We have made the suggested changes. | Abstract (Methods and Findings) |  |
| Abstract Conclusions:   3) Please address the implications of your study (as you have done in your Author Summary). | Thank you for the suggestion. We have added additional text to address the implications of the study in the Abstract Conclusions. | Abstract (Conclusion) |  |
| Introduction:   4) Line 118 - Please reference the three studies mentioned. | Thank you for the suggestion. We have referenced the three studies mentioned. | Introduction. |  |
| References:   5) Please ensure that journal name abbreviations match those found in the National Center for Biotechnology Information (NCBI) databases (http://www.ncbi.nlm.nih.gov/nlmcatalog/journals), and are appropriately formatted and capitalised. E.g. Ref 7 - Int J Gynaecol Obstet   Please also see https://journals.plos.org/plosmedicine/s/submission-guidelines#loc-references for further details on reference formatting. | Thank you for raising this error. We have reviewed the entire reference list and have ensured all references are appropriately formatted and abbreviated correctly in accordance with the National Center for Biotechnology Information (NCBI) databases. | References |  |
| **RESPONSE TO REVIEWER 1** | | |  |
| 1) Thanks authors for their effort to improve the manuscript. The extra sensitivity analysis on seasonal effect is adequate and well reported. The statistical aspects of the paper are satisfactory. No further issues needing attention. | Thank you for your comments and suggestions. |  |  |
